# The Pediatric Quality of Life Inventory 3.2 Diabetes Module: Preliminary Validation and Initial Evidence of Reliability and Factor Structure of the Croatian Version

**DOI:** 10.3390/children13070871

**Published:** 2026-06-29

**Authors:** Filip Petković, Zvonimir Užarević

**Affiliations:** Faculty of Education, University of Osijek, Cara Hadrijana 10, 31000 Osijek, Croatia; filip.petkovic4444@gmail.com

**Keywords:** PedsQL 3.2 diabetes module, type 1 diabetes mellitus, primary school children, Croatia

## Abstract

**Highlights:**

**What are the main findings?**

- This is the first study to examine preliminary validation of the Croatian version of the Pediatric Quality of Life Inventory 3.2 Diabetes Module.
- The preliminary results found that the instrument demonstrated internal consistency, test–retest reliability, and factor analysis supported the construct validity.

**What are the implications of the main findings?**

- The questionnaire enables standardized assessment of diabetes-related quality of life in Croatian-speaking children diagnosed with diabetes.
- The questionnaire supports routine clinical monitoring of diabetes-related quality of life, facilitates early identification of psychosocial challenges in children with diabetes, and enables cross-cultural comparison of pediatric diabetes outcomes.

**Abstract:**

Background/Objectives: Type 1 diabetes mellitus (T1DM) in primary school children presents unique challenges due to developmental dependence on adults, limited self-care abilities, and the need for continuous medical supervision. These factors may adversely affect health-related quality of life (HRQoL), particularly in early educational settings. Although diabetes-specific HRQoL assessment is essential for comprehensive pediatric diabetes care, no validated Croatian language instrument has previously been available. This preliminary study aimed to evaluate the initial validity, reliability, and factor structure of the Croatian version of the Pediatric Quality of Life Inventory (PedsQL) 3.2 Diabetes Module. Methods: This cross-sectional preliminary study was conducted in a clinical pediatric diabetes care setting in Croatia and included 70 children with T1DM, aged 7–14 years, and their parents or caregivers, recruited using convenience sampling. HRQoL was assessed using the Croatian versions of the PedsQL 4.0 Generic Core Scales and the PedsQL 3.2 Diabetes Module, which was translated using a forward-backward translation procedure. Reliability was evaluated using Cronbach’s α and test–retest reliability using the intraclass correlation coefficient (ICC). Construct validity was examined using Spearman’s correlation analysis and exploratory factor analysis. Results: Children with T1DM reported higher overall HRQoL than parent proxy reports, except for the Diabetes symptoms scale of the PedsQL 3.2 Diabetes Module and the School functioning scale of the PedsQL 4.0 Generic Core Scales. Internal consistency was satisfactory across all scales (Cronbach’s α = 0.71–0.85). Spearman’s correlations between subscales and total scores were strong (ρ = 0.61–0.92). Test–retest reliability was excellent (ICC = 0.982–0.996). Exploratory factor analysis supported construct validity: Bartlett’s test of sphericity was significant for both child (χ^2^ = 1398.57, *p* < 0.001) and parent reports (χ^2^ = 1302.74, *p* < 0.001), and the Kaiser–Meyer–Olkin measure indicated acceptable sampling adequacy (child = 0.65; parent = 0.68). Extracted factors explained 66.30% of the variance in child reports and 61.80% in parent reports, with factor loadings ranging from 0.41 to 0.89 and 0.41 to 0.85, respectively. Conclusions: The Croatian version of the PedsQL 3.2 Diabetes Module is an initial valid, reliable, and feasible instrument for assessing diabetes-specific HRQoL in Croatian primary school children with T1DM. Its use may support systematic HRQoL monitoring and improve family-centered pediatric diabetes care.

## 1. Introduction

Type 1 diabetes mellitus (T1DM) is one of the most common chronic endocrine diseases in childhood and represents a major public health concern due to its lifelong nature and increasing global incidence [1,2]. There is no definite opinion as to whether the increasing incidence of T1DM is due to improved recognition and diagnosis of affected children or simply an increase in incidence. According to the World Health Organization (WHO), in 2024, approximately 1.2 million people aged 0 to 20 years were diagnosed with type 1 diabetes [3]. For the same age group, the CroDiab national registry of people with diabetes recorded 2155 children and adolescents with T1DM in the Republic of Croatia in 2023 [4].

Although T1DM is commonly diagnosed in older primary school children and adolescents, a substantial proportion of cases occur during preschool and early primary school years, a period marked by rapid cognitive, emotional, and social development [5,6]. The diagnosis of T1DM at this age requires early initiation of lifelong disease management, including blood glucose monitoring, insulin administration, dietary regulation, and prevention of acute complications such as hypoglycemia and hyperglycemia [1,2]. Due to their limited ability to understand the disease and independently perform self-care activities, young children are highly dependent on parents, caregivers, and educational staff for daily diabetes management [6,7,8,9]. Consequently, successful integration of medical care into the school environment is essential to support both optimal disease control and the child’s psychosocial development, including participation in age-appropriate activities and peer interactions [5,8,9].

Health-related quality of life (HRQoL) is a multidimensional construct encompassing physical, emotional, and social well-being in the context of health and illness [10,11]. In pediatric populations, HRQoL assessment complements clinical indicators by capturing everyday functioning and psychosocial adaptation [12,13]. Research and assessment of HRQoL in the pediatric population are of great importance for developing policy measures and guidelines to improve the lives of this population, structuring treatment guidelines for specific diagnoses, and improving outcomes for individual children [14]. Almost half of the clinician researchers who addressed HRQoL changed their child’s therapy or treatment after receiving the results of a QoL assessment, and almost all researchers reported that the assessment results were useful and helpful [15]. The QoL related to children suffering from various chronic diseases, including T1DM, is a less frequently researched area compared to the healthy population of children and adolescents. It is also very important to use specific measuring instruments that cover different QoL aspects [16]. Speaking about diabetes-specific HRQoL, and therefore diabetes-specific HRQoL instruments, The Pediatric Quality of Life Inventory (PedsQL) 3.0 Diabetes Module stands for one of the most widely used instruments, and also an instrument with one of the largest numbers of international validations [17]. In 2018, an updated 3.2 version of this questionnaire was developed [18], and, similar to the 3.0 version, it is part of The Pediatric Quality of Life Inventory (PedsQL™), which is a modular instrument for measuring HRQoL in children and adolescents ages 2 to 18 [19]. In this study, we used and validated the Croatian version of the updated Diabetes Module 3.2 version of this diabetes-specific questionnaire.

Previous research indicates that children with T1DM may experience impairments in specific HRQoL domains, particularly those related to school functioning, emotional well-being, and social participation [20,21,22]. However, primary school children remain underrepresented in the literature despite the importance of early childhood for long-term development [5,23].

In Croatia, data on HRQoL among primary school children with T1DM are limited, and no validated Croatian diabetes-specific HRQoL instrument has previously been available. Although internationally developed instruments such as the PedsQL 3.2 Diabetes Module are widely used, cultural, linguistic, healthcare, and educational differences may influence how children and their parents perceive and report diabetes-related QoL. Therefore, cross-cultural validation is essential to ensure that the instrument accurately reflects the experiences and needs of Croatian children with T1DM. Accordingly, the aim of this preliminary study was to evaluate the initial evidence of validity, reliability, and factor structure of the Croatian version of the PedsQL 3.2 Diabetes Module, with particular emphasis on identifying HRQoL domains that may require targeted clinical and educational support.

## 2. Materials and Methods

### 2.1. Study Design and Participants

This preliminary cross-sectional study included a convenience sample of 70 primary school children diagnosed with T1DM (30 females and 40 males; age range 7–14 years, mean age 11.09 ± 2.30 years) and their parents or primary caregivers. Participants were recruited through Croatian associations of children with T1DM, through an invitation to participate in the study distributed by the associations. Inclusion criteria were a confirmed diagnosis of T1DM, primary school age (7–14 years), and the ability of both children and their parents or caregivers to understand and complete the Croatian language questionnaires. Children with cognitive impairments or other medical conditions that could substantially affect HRQoL independently of T1DM were excluded to reduce the influence of potential confounding factors. Parents/caregivers and their children independently completed the questionnaires after being informed about the aim and purpose of the research. By signing informed consent, parents/caregivers gave their consent to participate in the research, on behalf of the child and on their own behalf. Prior to participation and upon providing written informed consent, they were informed of their right to withdraw from the study at any time. Ethical approval for the study was granted by the Ethical Committee of the Faculty of Education, University of Osijek, Croatia (protocol code 602-04/25-04/04, 2158-63-02-25-20, 27 March 2025).

### 2.2. Assessment of Health-Related Quality of Life

In this preliminary research, the general and diabetes-specific HRQoL of the participants were assessed. For the general HRQoL, an already existing Croatian version of the PedsQL 4.0 Generic Core Scale was used [24]. For the diabetes-specific HRQoL of participants, the original PedsQL 3.2 Diabetes Module was translated into Croatian and, for the first time, validated in this study. In our preliminary research, we used versions of PedsQL 3.2 Diabetes Module for children aged from 5 to 7, from 8 to 12, and from 13 to 18. In all versions, the questionnaire contains child self-reports and parent proxy reports. Also, all age versions of the questionnaire consist of 33 items divided into 5 scales: ‘Diabetes symptoms’ (15 items), ‘Treatment barriers’ (5 items), ‘Treatment adherence’ (6 items), ‘Worry’ (3 items), and ‘Communication’ (4 items). The ‘Diabetes symptoms’ scale asks patients about the presence of general symptoms of the disease; the ‘Treatment barriers’ and ‘Treatment adherence’ scales focus on habits such as diet, insulin administration, and activities of daily living; the ‘Worry’ scale contains questions about concerns about crises and complications; and the ‘Communication’ scale refers to obstacles and difficulties in everyday communication. Each item in child self-reports and parent proxy reports consists of a 5-point Likert scale: 0—never; 1—almost never; 2—sometimes; 3—often; 4—almost always. The Likert scale is transformed into a scoring system from 0 to 100, where 0 = 100, 1 = 75, 2 = 50, 3 = 25, and 4 = 0. The total score is calculated by the sum of the points achieved on all items divided by the number of items, with a higher score indicating better QoL. In addition to the overall score, the questionnaire can sometimes be divided into two parts or two summary scores: ‘Diabetes symptoms summary score’ and ‘Diabetes management summary score’. ‘Diabetes symptoms summary score’ consists of 15 items contained in the subscale ‘Diabetes symptoms’, and ‘Diabetes management summary score’ consists of 18 items, or the total sum of items in the subscales ‘Treatment barriers’ (5 items), ‘Treatment adherence’ (6 items), ‘Worry’ (3 items), and Communication (4 items). In the standard version to be used in the preliminary study, all questions refer to the period of the last month [18]. The questionnaire is widely used, and in addition to the original version being validated in several cycles on different population groups, including participants with both type 1 and type 2 diabetes separately or together [18,25,26,27,28], the questionnaire has so far been translated and validated on a population suffering from T1DM in Korea [29] and India [30]. Permission for the validation of the original questionnaire into Croatian was obtained from the original developer—MAPI Research Trust (at https://www.pedsql.org/translations.html, accessed on 1 May 2026). The translation process followed a double-blinded forward–backward procedure. The aforementioned translation and adaptation procedure is described in detail in the papers by Guillemin et al. [31] and Beaton et al. [32].

### 2.3. Statistical Analysis

The required sample size was calculated using G-power software based on an effect size of 0.50, a significance level of 0.05, and a statistical power of 0.80. Data were entered into Microsoft Office Excel and analyzed using Statistica (TIBCO, Palo Alto, CA, USA). Descriptive statistics were used to summarize participant characteristics and questionnaire scores, and the Shapiro–Wilk test was used to assess data distribution normality. The internal consistency of the PedsQL 3.2 Diabetes Module scales and the total score was evaluated using Cronbach’s α coefficient, with values ≥0.70 considered indicative of acceptable internal consistency. Test–retest reliability was assessed using the intraclass correlation coefficient (ICC) with 95% confidence intervals (95% CI), where values ≥ 0.80 indicated excellent reproducibility. Convergent validity was examined using Spearman’s correlation coefficients to assess the relationship between individual scale scores and the total score. Construct validity was evaluated using exploratory factor analysis (EFA) with varimax rotation. The suitability of the data for EFA was determined using the Kaiser–Meyer–Olkin (KMO) measure of sampling adequacy, with values ≥0.60 considered acceptable, and Bartlett’s test of sphericity (BTS) with statistical significance indicating that the correlation matrix was appropriate for factor analysis. Factor extraction was based on eigenvalues ≥1 and factor loadings ≥0.40 [33,34,35]. Statistical significance was set at *p* < 0.05.

## 3. Results

Our preliminary research included 70 primary school children with T1DM and their parents or caregivers who were recruited from associations of children with T1DM in Croatia. Table 1 shows demographic and clinical characteristics of the participants.

As the main questionnaire in our research for diabetes-specific HRQoL of participants, we used and validated the Croatian version of the PedsQL 3.2 Diabetes Module. Descriptive statistics, internal consistency, and correlations for the scales and total scores for the child self-report and parent proxy report versions of the questionnaire are listed in Table 2.

As the second questionnaire, and for the purpose of assessing participants’ general HRQoL, we used the already existing and validated Croatian version of the PedsQL 4.0 Generic Core scale. Table 3 shows correlations between scales and total scores of the two mentioned questionnaires.

In the process of validation of the Croatian version of the PedsQL 3.2 Diabetes Module questionnaire, we performed test–retest reliability to evaluate consistency and stability. The results are listed in Table 4.

As another part of the validation process of the questionnaire, we also decided to assess the strength and direction of the relationships between the questionnaire scales and to examine the factor structure. For child self-reports, BTS indicated a high and significant correlation (χ^2^ = 1398.57, *p* < 0.001). The KMO value measured 0.65. The factors extracted accounted for 66.30% of the total variance, with factor loadings ranging from 0.41 to 0.89 (Table 5).

Regarding parent proxy reports, BTS indicated a high and significant correlation (χ^2^ = 1302.74, *p* < 0.001). The KMO value measured 0.68. The factors extracted accounted for 61.80% of the total variance, with factor loadings ranging from 0.41 to 0.85 (Table 6).

To strengthen the results, we also compared associations between child self-reports and parent proxy reports and participants’ clinical characteristics, including glycated hemoglobin (HbA1c), body mass index (BMI), and duration of diabetes. No statistical correlation was found; precise results are shown in Table 7.

## 4. Discussion

To the best of our knowledge, this is the first preliminary study in Croatia which investigates diabetes-specific HRQoL in patients suffering from T1DM, in which the Croatian version of the PedsQL 3.2 Diabetes Module was used for HRQoL assessment.

We found it necessary and important to investigate the HRQoL of primary school children suffering from T1DM and include both children and their parents, because of the mentioned and proven statement that disease management in this age group relies almost entirely on parents and caregivers, placing a considerable burden on families and requiring constant supervision [6,7,9].

The reason for translating and validating the Croatian version of the PedsQL 3.2 Diabetes Module rather than using already translated and clinically implemented questionnaires available in Croatia is that the PedsQL 3.2 Diabetes Module is a rating scale specifically designed for quick screening, which facilitates clinical management and enables evaluation of treatment effects [18].

In our research, primary school children with T1DM in Croatia reported high general and diabetes-specific HRQoL, indicating that early diagnosis and modern disease management can preserve general well-being [5,17]. This finding aligns with prior research suggesting that structured parental involvement, adherence to insulin regimens, and close collaboration with healthcare providers contribute to maintaining physical, emotional, and social functioning even at a young age [7,9].

In our preliminary study, primary school children with T1DM reported higher overall general and diabetes-specific HRQoL than their parents, which is consistent with previous findings suggesting that young children often perceive their condition as less burdensome than their caregivers. This discrepancy may be explained by developmental factors, as younger children are less aware of the long-term consequences of T1DM and are largely protected from disease-related responsibilities by parental involvement. In contrast, parents may perceive greater impairment due to their awareness of potential complications, continuous responsibility for disease management, and concerns about their child’s future health.

The lower scores reported in the Diabetes symptoms domain by both children and parents indicate that the physical manifestations of T1DM and daily symptom management remain a significant challenge despite overall good adaptation. Additionally, lower scores in the school functioning domain emphasize that the school environment may be a vulnerable setting, where diabetes management, participation in academic activities, and social integration require additional support and collaboration between families, educators, and healthcare professionals.

The finding that emotional functioning was the lowest-rated domain of generic HRQoL among both children and parents highlights the psychological burden associated with living with a chronic condition during an important developmental period. Children with T1DM may experience fears related to blood glucose fluctuations, medical procedures, or feeling different from their peers, while parents may recognize emotional difficulties associated with maintaining normal childhood experiences under the constraints of a chronic illness. Conversely, high scores in the social functioning domain suggest that, with appropriate family support and successful adaptation, children with T1DM can maintain satisfying peer relationships and participate in age-appropriate social activities.

The high ratings of the treatment adherence domain may reflect the central role of parents and caregivers in organizing daily diabetes management, including insulin administration, dietary regulation, and glucose monitoring. Consistent family routines, parental supervision, and effective communication with healthcare professionals may facilitate treatment adherence and contribute to a generally positive perception of HRQoL in young children with T1DM [6,7,9].

Research focusing specifically on HRQoL among primary school children with T1DM remains limited, and findings for this age group are often combined with data from older children and adolescents, making age-specific interpretation challenging. Furthermore, studies using the PedsQL 3.2 Diabetes Module with both child self-reports and parent proxy reports are relatively scarce, limiting direct comparison with the existing literature. Nevertheless, the limited availability of comparable studies highlights the importance of generating age-specific and culturally relevant data on HRQoL in young children with T1DM.

Beyond comparison with previous validation studies, the present findings have important implications for pediatric diabetes care. The use of a validated diabetes-specific HRQoL instrument enables healthcare professionals to systematically identify domains in which children experience difficulties, such as emotional well-being, diabetes-related symptoms, and challenges in the school environment. Regular assessment of both child and parent perspectives may facilitate more individualized interventions, strengthen family-centered diabetes care, and support collaboration between healthcare providers, parents, and educational staff. Therefore, integrating HRQoL monitoring into routine clinical practice may contribute not only to improved psychosocial adaptation but also to more comprehensive management of T1DM during a critical developmental period [29,30].

In an original validation study conducted in 2018 by Varni et al. [18] on participants with T1DM, the validity, reliability, and factor structure of the PedsQL 3.2 Diabetes module were tested in children with T1DM and their parents. Their sample of participants was older (14.25 ± 3.6 years), had longer disease duration (5.4 ± 3.9 years), higher HbA1c (8.8 ± 1.9), and higher BMI (22.5 ± 6.4 kg/m^2^). Factor loading for child self-reports in their research ranged from 0.37 to 0.80 for ‘Diabetes symptoms summary score’ and from 0.31 to 0.79 for ‘Diabetes management summary score’. In both summary scores, those results are lower than those in our research. On the other hand, in terms of internal consistency, Cronbach’s α coefficients were higher than those in our study. Cronbach’s α coefficients in the original validation study were 0.88 (‘Diabetes symptoms’) and 0.89 (‘Diabetes management’) for child self-reports, while for parent proxy reports, they measured 0.90 for both ‘Diabetes symptoms’ and ‘Diabetes management’ summary scores. When it comes to intercorrelations of scales and total scores between the PedsQL 3.2 Diabetes Module and the PedsQL 4.0 Generic Core scales, Varni et al. measured higher correlation coefficients between the total score of the PedsQL 4.0 Generic Core scale and ‘Diabetes symptoms’ and ‘Diabetes management’ summary scores for both child self-reports and parent-proxy reports. In their results, as the most significant correlation of sample characteristics with ‘Diabetes symptoms’ and ‘Diabetes management’ summary scores, Varni et al. point out the HbA1c value [18]. In our results, although no significant correlations were found for any clinical characteristic, the highest result was observed for T1DM duration in parent proxy reports (ρ = −0.12), indicating that the longer their child has had T1DM, the lower diabetes-specific QoL is reported by their parent. That is quite logical since there is proven psychological burden in parents as disease duration increases and progresses [6,7,9].

Boo et al. assessed diabetes-specific HRQoL of Korean children and adolescents with T1DM using the PedsQL 3.2 Diabetes Module. Their sample included only children with T1DM and was older (13.3 ± 2.8 years), had a shorter disease duration (4.0 ± 3.3 years), and a higher HbA1c level (8.8 ± 1.8) compared with our sample. The total score for child self-reports in their study was 69.2 ± 14.1, indicating that children in our study estimated their diabetes-specific HRQoL to be better and higher. Cronbach’s α for the Korean version of the PedsQL 3.2 Diabetes Module ranged from 0.66 to 0.88, which also represents lower internal consistency than that we measured [29].

In 2021, Bhavani et al. assessed HRQoL in children with T1DM in India aged 18 years or younger using the PedsQL Diabetes 3.2 Module and the PedsQL 4.0 Generic Core scales. Their sample was younger (8.2 ± 4.6 years), had a longer duration of disease (8.2 ± 5.6 years), and a higher HbA1c value (8.7) when compared to our participants. Both general and diabetes-specific HRQoL were estimated to be lower in both child self-reports (general—77.23; diabetes-specific—68.15) and parent proxy reports (general—71.23; diabetes-specific—64.35) than in our research. Regarding the clinical and demographic characteristics of participants, Bhavani et al. concluded that HbA1c had a negative correlation with emotional scoring and a positive correlation with treatment adherence [30].

This preliminary study has several limitations that should be considered when interpreting the findings. First, the cross-sectional design prevents conclusions regarding causal relationships or changes in general and diabetes-specific HRQoL over time. Second, the relatively small sample size and convenience sampling through associations of children with T1DM may limit the generalizability of the findings and introduce potential selection bias, as families actively involved in patient associations may differ from the broader population of children with T1DM. Although small sample sizes are common in psychosocial studies involving pediatric clinical populations [36], the participant-to-item ratio in the present preliminary study was below the thresholds generally recommended for comprehensive psychometric validation and factor analytic procedures. Therefore, the observed two-factor structure should be interpreted as a preliminary, hypothesis-generating finding rather than definitive evidence of the underlying structure of the Croatian version of the PedsQL 3.2 Diabetes Module. Future studies involving larger and more representative samples are needed to replicate the factor structure using confirmatory factor analysis (CFA) and to provide additional evidence of the instrument’s psychometric properties. Furthermore, this preliminary study did not include a healthy control group, which limits the ability to assess known-groups validity and determine whether the instrument can effectively distinguish between HRQoL perceptions of children with T1DM and those of healthy peers. Future studies should include age- and sex-matched healthy control participants to provide a broader understanding of the disease-specific impact of T1DM on HRQoL. Additionally, potential factors known to influence HRQoL, such as diabetes duration, metabolic control, socioeconomic background, comorbid conditions, family functioning, and availability of educational support, were not examined and may have affected the reported HRQoL outcomes. Finally, although parent proxy reports provide valuable complementary information, they may not fully reflect children’s subjective experiences and perceptions of living with T1DM. Future longitudinal and multicenter studies involving larger and more diverse samples are warranted to further evaluate the instrument’s psychometric performance, including its responsiveness to change over time, discriminant validity, and applicability across different clinical and social contexts.

## 5. Conclusions

The Croatian version of the PedsQL 3.2 Diabetes Module shows promising preliminary evidence of initial reliability and validity as a diabetes-specific HRQoL assessment tool for primary school children with T1DM. Differences between child and parent perceptions underscore the importance of considering both perspectives when assessing HRQoL in pediatric diabetes care. Although the instrument may be a useful tool for supporting the identification of emotional, school-related, and diabetes-specific challenges in clinical practice, the present preliminary findings should be considered hypothesis-generating and require confirmation in larger and more diverse samples, including further evaluation of the observed factor structure using CFA.

## Figures and Tables

**Table 1:** Demographic and clinical characteristics of participants.

Characteristic	N (%) or MV ± SD
Total N of participants	70
Gender (male/female)	30 (42.86)/40 (57.14)
Age (years)	11.09 ± 2.30
Duration of diabetes (years)	4.51 ± 2.99
HbA1c (%)	7.04 ± 1.40
BMI (kg/m^2^)	19.36 ± 3.73
BMI (percentile)	62.18 ± 27.65

N—number; MV—mean value; SD—standard deviation; HbA1c—glycated hemoglobin; BMI—body mass index.

**Table 2:** Descriptive statistics, internal consistency, and correlations for the PedsQL 3.2 Diabetes Module scales and total scores in child self-reports and parent proxy reports.

Scales (N of Items)	MV ± SD	Median (IQR)	Min	Max	Cronbach’s α	ρ
Child self-report						
Diabetes symptoms (15)	67.71 ± 15.96	75 (50)	25	100	**0.82**	0.86 *
Treatment barriers (5)	74.64 ± 18.60	75 (25)	0	100	**0.80**	0.70 *
Treatment adherence (6)	81.55 ± 14.43	100 (25)	50	100	**0.78**	0.81 *
Worry (3)	68.69 ± 20.78	50 (25)	0	100	**0.85**	0.66 *
Communication (4)	77.68 ± 20.18	87.50 (37.50)	12.50	100	**0.79**	0.73 *
Diabetes management (18) ^a^	76.63 ± 14.41	87.50 (25)	25	100	**0.74**	0.91 *
Total score (33)	72.58 ± 13.40	75 (43.75)	50	100	**0.83**	**-**
Parent proxy report						
Diabetes symptoms (15)	68.17 ± 13.39	75 (43.75)	25	100	**0.76**	0.81 *
Treatment barriers (5)	72.43 ± 19.33	75 (50)	0	100	**0.76**	0.75 *
Treatment adherence (6)	78.21 ± 16.71	100 (34.38)	25	100	**0.71**	0.83 *
Worry (3)	66.43 ± 18.93	75 (25)	0	100	**0.79**	0.61 *
Communication (4)	73.30 ± 23.41	75 (50)	0	100	**0.76**	0.66 *
Diabetes management (18) ^a^	73.55 ± 15.09	75 (37.50)	25	100	**0.72**	0.92 *
Total score (33)	71.10 ± 12.58	75 (43.75)	50	100	**0.77**	**-**

N—number; MV—mean value; SD—standard deviation; IQR—interquartile range; Min—minimum score; Max—maximum score; ρ—Spearman’s correlation coefficient; ^a^—diabetes management scale includes treatment barriers, treatment adherence, worry, and communication scales; *—statistically significant correlation at *p* < 0.05 (marked in bold).

**Table 3:** Correlations of PedsQL 3.2 Diabetes Module scales and total scores with PedsQL 4.0 Generic Core scales and total scores in child self-reports and parent proxy reports.

	PedsQL 4.0 Generic Core Scales and Total Scores (N of Items)					
PedsQL 3.2 Diabetes Module Scales and Total Scores (N of Items)	Physical Functioning (8)	Emotional Functioning (5)	Social Functioning (5)	School Functioning (5)	Psychosocial Functioning (15) ^b^	Total Score (23)
	MV ± SD					
Child self-reports	83.62 ± 11.77	66.79 ± 19.71	85.57 ± 17.02	72.86 ± 18.87	75.07 ± 14.41	78.04 ± 12.64
Diabetes symptoms (15)	ρ = 0.43 *	ρ = 0.33 *	ρ = 0.41 *	ρ = 0.39 *	ρ = 0.47 *	ρ = 0.48 *
Diabetes management (18) ^a^	ρ = 0.40 *	ρ = 0.44 *	ρ = 0.37 *	ρ = 0.39 *	ρ = 0.46 *	ρ = 0.48 *
Total score (33)	ρ = 0.46 *	ρ = 0.44 *	ρ = 0.44 *	ρ = 0.44 *	ρ = 0.52 *	ρ = 0.54 *
Parent proxy reports	80.98 ± 13.15	62.86 ± 20.35	84.14 ± 18.00	74.21 ± 19.10	73.74 ± 15.38	76.26 ± 12.52
Diabetes symptoms (15)	ρ = 0.29 *	ρ = 0.25 *	ρ = 0.26 *	ρ = 0.42 *	ρ = 0.37 *	ρ = 0.35 *
Diabetes management (18) ^a^	ρ = 0.25 *	ρ = 0.25 *	ρ = 0.41 *	ρ = 0.31 *	ρ = 0.38 *	ρ = 0.32 *
Total score (33)	ρ = 0.26 *	ρ = 0.29 *	ρ = 0.39 *	ρ = 0.42 *	ρ = 0.45 *	ρ = 0.40 *

N—number; MV—mean value; SD—standard deviation; ^a^—Diabetes management scale includes treatment barriers, treatment adherence, worry, and communication scales; ^b^—psychosocial functioning scale includes emotional, social, and school functioning scales; ρ—Spearman’s correlation coefficient; *—statistically significant correlation at *p* < 0.05.

**Table 4:** The test–retest reliability of the PedsQL 3.2 Diabetes Module scales and total scores for child self-reports and parent proxy reports.

Scales (N of Items)	Test MV ± SD	Retest MV ± SD	ICC (95% CI)
Child self-reports			
Diabetes symptoms (15)	67.71 ± 15.96	69.22 ± 15.78	0.992 (0.987–0.995)
Treatment barriers (5)	74.64 ± 18.60	78.05 ± 15.46	0.993 (0.989–0.996)
Treatment adherence (6)	81.55 ± 14.43	83.11 ± 12.44	0.984 (0.974–0.990)
Worry (3)	68.69 ± 20.78	70.34 ± 21.42	0.993 (0.990–0.996)
Communication (4)	77.68 ± 20.18	80.02 ± 16.00	0.990 (0.984–0.994)
Diabetes management (18) ^a^	76.63 ± 14.41	79.58 ± 10.92	0.982 (0.971–0.989)
Total score (33)	72.58 ± 13.40	74.31 ± 11.83	0.983 (0.972–0.989)
Parent proxy reports			
Diabetes symptoms (15)	68.17 ± 13.39	68.06 ± 11.59	0.988 (0.980–0.992)
Treatment barriers (5)	72.43 ± 19.33	69.85 ± 18.01	0.996 (0.993–0.997)
Treatment adherence (6)	78.21 ± 16.71	75.92 ± 13.78	0.992 (0.988–0.995)
Worry (3)	66.43 ± 18.93	63.55 ± 16.67	0.993 (0.988–0.995)
Communication (4)	73.30 ± 23.41	71.23 ± 20.95	0.992 (0.986–0.995)
Diabetes management (18) ^a^	73.55 ± 15.09	70.87 ± 13.10	0.991 (0.986–0.995)
Total score (33)	71.10 ± 12.58	68.98 ± 10.67	0.982 (0.971–0.988)

N—number; MV—mean value; SD—standard deviation; ICC—intra-class correlation coefficient; CI—confidence interval; ^a^—diabetes management scale includes treatment barriers, treatment adherence, worry, and communication scales.

**Table 5:** Factor loadings of the PedsQL 3.2 Diabetes Module for child self-reports.

Factor Structure			
Scales	Items	Diabetes Symptoms	Diabetes Management
Diabetes symptoms	I feel hungry	0.60	
	I feel thirsty	0.71	
	I have to go to the bathroom too often	0.62	
	I have stomachaches	0.66	
	I have headaches	0.69	
	I feel like I need to throw up	0.73	
	I go ‘low’	0.60	
	I go ‘high’	0.49	
	I feel tired	0.52	
	I get shaky	0.71	
	I get sweaty	0.63	
	I feel dizzy	0.47	
	I feel weak	0.68	
	I have trouble sleeping	0.65	
	I get cranky or grumpy	0.43	
Treatment barriers	It hurts to get my finger pricked		0.71
	It hurts to get insulin shots		0.84
	I am embarrassed by my diabetes treatment		0.83
	My parents and I argue about my diabetes care		0.83
	It is hard for me to do everything I need to do to care for my diabetes		0.78
Treatment adherence	It is hard for me to take blood glucose tests		0.49
	It is hard for me to take insulin shots		0.78
	It is hard for me to exercise or do sports		0.49
	It is hard for me to keep track of carbohydrates		0.66
	It is hard for me to carry a fast-acting carbohydrate		0.41
	It is hard for me to snack when I go ‘low’		0.70
Worry	I worry about going ‘low’		0.69
	I worry about going ‘high’		0.83
	I worry about long-term complications from diabetes		0.49
Communication	It is hard for me to tell the doctors and nurses how I feel		0.74
	It is hard for me to ask the doctors and nurses questions		0.89
	It is hard for me to explain my illness to other people		0.42
	I am embarrassed about having diabetes		0.75

**Table 6:** Factor loadings of the PedsQL 3.2 Diabetes Module for parent proxy reports.

Factor Structure			
Scales	Items	Diabetes Symptoms	Diabetes Management
Diabetes symptoms	Feeling hungry	0.56	
	Feeling thirsty	0.52	
	Having to go to the bathroom too often	0.46	
	Having stomachaches	0.59	
	Having headaches	0.79	
	Feeling like they need to throw up	0.85	
	Going ‘low’	0.48	
	Going ‘high’	0.41	
	Feeling tired	0.69	
	Getting shaky	0.74	
	Getting sweaty	0.61	
	Feeling dizzy	0.75	
	Feeling weak	0.63	
	Having trouble sleeping	0.61	
	Getting cranky or grumpy	0.42	
Treatment barriers	Finger prick causing them pain		0.67
	Insulin shots causing them pain		0.72
	Getting embarrassed about their diabetes treatment		0.61
	Arguing with me or my spouse about diabetes care		0.66
	It is hard for my child/teen to do everything they need to do to care for their diabetes		0.69
Treatment adherence	It is hard for my child/teen to take blood glucose tests		0.47
	It is hard for my child/teen to take insulin shots		0.81
	It is hard for my child/teen to exercise or do sports		0.62
	It is hard for my child/teen to track carbohydrates		0.63
	It is hard for my child/teen to carry a fast-acting carbohydrate		0.70
	It is hard for my child/teen to snack when they go ‘low’		0.44
Worry	Worrying about going ‘low’		0.70
	Worrying about going ‘high’		0.85
	Worrying about long-term complications from diabetes		0.57
Communication	Telling the doctors and nurses how they feel		0.72
	Asking the doctors and nurses questions		0.67
	Explaining their illness to other people		0.48
	Getting embarrassed about having diabetes		0.85

**Table 7:** Associations of PedsQL 3.2 Diabetes Module total scores with clinical characteristics.

PedsQL 3.2 Diabetes Module Total Scores	HbA1c (%)	BMI (Percentile)	Duration of Diabetes (Years)
Child self-reports	ρ = −0.01	ρ = 0.14	ρ = −0.05
Parent proxy reports	ρ = −0.03	ρ = −0.07	ρ = −0.12

HbA1c—glycated hemoglobin; BMI—body mass index; ρ—Spearman’s correlation coefficient.

## Data Availability

The data presented in this study are available on request from the corresponding author.
